# Work time pressure, work–family conflict, sleep quality, and burnout among intensive care nurses: a cross-sectional study using structural equation modeling

**DOI:** 10.3389/fpubh.2026.1803875

**Published:** 2026-05-25

**Authors:** Dongmin Wang, Lili Li, Ke Xu, Xiaofeng Su, Dan Liao, Xu Liu, Yan Jiang

**Affiliations:** 1 West China School of Nursing, Sichuan University/Department of Nursing, West China Hospital, Sichuan University, Chengdu, China; 2 Evidence-based Nursing Center, West China Hospital, Sichuan University, Chengdu, China; 3 Sichuan Provincial Engineering Research Center of Medical Nursing Equipment and Materials, Chengdu, China; 4 Department of Medicine, Northwest Minzu University, Lanzhou, China; 5 The People’s Liberation Army Joint Logistic Support Force Sanya Rehabilitation and Recuperation Center, Sanya, China; 6 Department of Nursing, West China Hospital, Sichuan University/West China School of Nursing, Sichuan University, Chengdu, China

**Keywords:** burnout, intensive care unit nurses, sleep quality, work time pressure, work–family conflict

## Abstract

**Background:**

Burnout is highly prevalent among intensive care unit (ICU) nurses and poses a serious threat to workforce stability, nurses’ well-being, and patient safety. Work time pressure has been identified as a key occupational stressor in critical care settings; however, the mechanisms through which work time pressure contributes to burnout among ICU nurses remain insufficiently understood. Clarifying these mechanisms may help identify potential targets for burnout prevention in ICU settings.

**Objective:**

This study aimed to examine the association between work time pressure and burnout among ICU nurses and to explore the mediating roles of work–family conflict and sleep quality in this relationship.

**Methods:**

A cross-sectional survey was conducted among 545 ICU nurses from four hospitals in Chengdu, Lanzhou and Sanya, China. Data were collected using validated self-report questionnaires measuring time pressure, work–family conflict, sleep quality, and burnout. Structural equation modeling (SEM) was employed to test the hypothesized mediation model. Indirect effects were examined using a bootstrapping procedure with 5,000 resamples.

**Results:**

Work time pressure was positively associated with burnout among ICU nurses. Work–family conflict and sleep quality each independently mediated the relationship between work time pressure and burnout. In addition, a significant serial mediating effect was observed, indicating that higher time pressure was associated with increased work–family conflict, which in turn was related to poorer sleep quality and subsequently higher levels of burnout.

**Conclusion:**

Work time pressure is an important risk factor for burnout among ICU nurses. Work–family conflict and sleep quality play both independent and sequential mediating roles in this relationship. These findings suggest that reducing work-related time pressure, supporting work–family balance, and promoting healthy sleep may contribute to burnout prevention among ICU nurses.

## 1 Introduction

Burnout is a psychological syndrome characterized by emotional exhaustion, depersonalization, and a reduced sense of personal accomplishment, resulting from prolonged exposure to chronic stressors (1, 2). In nursing populations, burnout not only impairs individual physical and mental health but is also closely associated with decreased job performance, increased turnover intention, compromised patient safety, and poorer quality of care (3). Especially in high-intensity clinical environments such as intensive care units (ICUs), nurses are required to continuously manage critically ill patients, rapidly changing conditions, complex technologies, and life-threatening emergencies (4). These demands require sustained vigilance, rapid decision-making, and emotional labor, placing ICU nurses at particularly high risk for burnout (5). Therefore, burnout among ICU nurses has become a critical issue affecting both workforce sustainability and the stability of healthcare systems.

However, a global and persistent challenge confronting healthcare systems is the shortage of nurses (6). The growing gap between increasing healthcare demands and limited nursing human resources has led to heavier workloads, extended working hours, and intensified work time pressure (7). ICU nurses are especially affected, as critical care units operate continuously and depend on highly specialized nursing labor (7). High workload, frequent night shifts, long working hours, and unpredictable clinical events contribute to cumulative stress and insufficient recovery. As a result, ICU nurses report significantly higher levels of burnout, fatigue, and intention to leave compared with nurses in general wards (8). This trend not only threatens the retention of experienced ICU nurses but also directly affects patient safety and quality of critical care delivery (8). Therefore, identifying modifiable occupational stressors and clarifying their internal mechanisms in relation to burnout has become a central concern in nursing management and occupational health research.

Among the numerous occupational stressors, work time pressure has gradually attracted attention (9) work time pressure refers to individuals’ subjective perception that available working time is insufficient to complete required tasks and cope with work demands (10). In ICU settings, nurses must simultaneously manage high patient acuity, urgent clinical interventions, complex monitoring tasks, and extensive documentation within limited time frames (11). Persistent time pressure has been shown to be associated with emotional exhaustion, impaired recovery, and reduced well-being (12). However, existing studies have mainly focused on general workload or shift patterns, while the specific role of perceived time pressure and its underlying transmission mechanisms toward burnout among ICU nurses remain insufficiently explored (13). In addition, burnout does not develop solely through direct exposure to work stressors. According to occupational stress and conservation of resources theories, work stress often affects individuals through disruptions in work–life balance and recovery processes (14). Work–family conflict reflects a form of inter-role conflict in which work demands interfere with family responsibilities (15), while sleep quality represents a core physiological recovery process essential for emotional regulation and stress restoration (16, 17). Both factors have been independently associated with burnout among nurses (18). Nevertheless, few studies have simultaneously integrated work time pressure, work–family conflict, and sleep quality into a unified explanatory framework, particularly among ICU nurses (19, 20). The potential chain mechanisms linking work time pressure to burnout through psychosocial conflict and impaired sleep remain unclear.

Therefore, this study aims to clarify the internal relationship and interaction mechanisms among work time pressure, work–family conflict, sleep quality, and burnout among ICU nurses, in order to provide a theoretical basis for targeted organizational interventions and workforce management strategies.

## 2 Background

### 2.1 Work time pressure and burnout

According to occupational stress theory, excessive job demands that exceed individuals’ available resources are key antecedents of burnout (21). Work time pressure represents a core form of job demand, reflecting the mismatch between task requirements and available time (9). When employees consistently perceive insufficient time to complete work tasks, they are more likely to experience sustained psychological tension, emotional depletion, and reduced professional efficacy (22). This mechanism can be further understood within the framework of the Job Demands–Resources (JD–R) model, which posits that job demands requiring sustained effort—such as heavy workload and work time pressure-consume individuals’ physical and psychological resources and may initiate a health impairment process that ultimately results in burnout (23). Within this theoretical perspective, work time pressure can be conceptualized as a salient job demand that increases vulnerability to burnout among healthcare professionals.

In ICU environments, work time pressure is intensified by high patient severity, frequent emergencies, and constant interruptions (5). ICU nurses must prioritize life-saving tasks, coordinate multidisciplinary care, and respond rapidly to clinical deterioration, often under strict time constraints (13). Prolonged exposure to such work time pressure may lead to emotional exhaustion, frustration, and a diminished sense of professional accomplishment, which are central components of burnout (24). Although prior research has shown that workload and time-related stress are associated with nurse burnout, most studies have examined general nursing populations (25, 26), with limited attention to ICU nurses and to the specific construct of work time pressure. Based on these considerations, we propose Hypothesis 1:

*H1*: Work time pressure positively predicts burnout among ICU nurses.

### 2.2 The potential mediating role of work–family conflict

According to the Spillover Crossover Model, a stressful work environment can disrupt individuals’ role balance and increase work–family conflict (27). When work demands exceed available time and energy resources, individuals are more likely to experience role overload, in which work responsibilities intrude into family life (27). Work time pressure represents a core form of job demand, reflecting the perception that available working time is insufficient to complete required tasks (15). Persistent exposure to such pressure not only heightens psychological tension but also compels individuals to invest additional temporal and emotional resources into work, thereby reducing their capacity to fulfill family roles (28). Previous studies have shown that long working hours, high workload, and time-related stressors are significantly associated with elevated levels of work–family conflict (29, 30).

Additionally, a growing body of evidence indicates that work–family conflict is closely associated with burnout and plays a critical mediating role between occupational stressors and adverse psychological outcomes (31). Nurses experiencing high work–family conflict are more likely to report emotional exhaustion, psychological distress, and reduced well-being (15). In high-intensity healthcare environments, work–family conflict has been identified as an important mechanism linking workload and shift-related stress to burnout (15). In the ICU context, nurses are frequently exposed to extended shifts, unpredictable emergencies, and high emotional demands, which substantially increase the risk of conflicts between work and family roles (13). When work persistently interferes with family life, emotional resources are gradually depleted, thereby accelerating the development of burnout (13). Therefore, this study hypothesizes that work time pressure may increase ICU nurses’ work–family conflict, which in turn contributes to burnout. Based on the above discussion, we propose Hypothesis 2:

*H2*: Work–family conflict mediates the relationship between work time pressure and burnout among ICU nurses.

### 2.3 The potential mediating role of sleep quality

Sleep quality reflects individuals’ overall subjective evaluation of sleep duration, continuity, and restorative effectiveness, and represents a core physiological resource for recovery from work-related stress (16). According to the conservation of resources theory, adequate and high-quality sleep is essential for restoring depleted physical and psychological resources (32), whereas persistent work stress may impair sleep processes, leading to cumulative fatigue and emotional dysregulation (33). Work time pressure, as a chronic occupational stressor, often extends working hours, compresses rest time, and increases cognitive and emotional arousal after work, thereby disrupting normal sleep patterns (33). Previous studies have shown that high job demands, long working hours, and time-related stressors are significantly associated with poor sleep quality, insomnia symptoms, and circadian rhythm disturbances among nurses (17, 34, 35).

Furthermore, substantial evidence indicates that impaired sleep quality is closely linked to burnout (36). Poor sleep weakens individuals’ capacity for emotional regulation, stress tolerance, and cognitive functioning, thereby increasing vulnerability to emotional exhaustion, depersonalization, and reduced personal accomplishment (36). In nursing populations, sleep disturbances have been consistently associated with fatigue, reduced work efficiency, and higher levels of burnout (37). In the ICU context, nurses are particularly susceptible to sleep problems due to rotating shifts, night work, exposure to critical incidents, and sustained hyperarousal (33). This study therefore posits that work time pressure may undermine ICU nurses’ sleep quality, which in turn accelerates the development of burnout. Based on the above discussion, we propose Hypothesis 3:

*H3*: Sleep quality mediates the relationship between work time pressure and burnout among ICU nurses.

### 2.4 The relationship between work–family conflict and sleep quality

Work–family conflict and sleep quality represent two interrelated but conceptually distinct components of the stress-recovery process (15, 36). Work–family conflict reflects individuals’ cognitive and emotional appraisal of incompatibility between work and family roles, whereas sleep quality reflects the effectiveness of physiological and psychological recovery (15, 36). Although different in nature, both constructs are closely linked to stress regulation and resource restoration. Accumulating evidence indicates that higher levels of work–family conflict are associated with increased sleep disturbances, including difficulty initiating and maintaining sleep, shortened sleep duration, and poorer subjective sleep quality (38). Persistent role conflict may prolong psychological activation and emotional strain, thereby disrupting pre-sleep relaxation and impairing sleep processes (38). Among nurses, particularly in high-intensity settings, work–family conflict has been shown to significantly predict sleep problems (39). In the ICU context, unresolved role conflicts and emotional demands may continue after work, increasing rumination and physiological arousal and further undermining sleep quality (8). From a theoretical perspective, this sequential relationship is consistent with occupational stress frameworks suggesting that job demands initiate strain processes that subsequently interfere with recovery mechanisms (21). Work time pressure, as a primary job demand, may first generate role-related strain in the form of work–family conflict, which then impairs sleep as a key recovery resource. Impaired sleep, in turn, may reduce resource replenishment and increase vulnerability to burnout.

Integrating these considerations into the proposed framework, this study posits that work time pressure may increase work–family conflict, which subsequently impairs sleep quality and contributes to burnout. Therefore, we propose:

*H4*: work–family conflict and sleep quality play a serial mediating role in the relationship between work time pressure and burnout among ICU nurses.

## 3 Methods

### 3.1 Design and participants

A cross-sectional survey design was employed in this study, which was conducted and reported in accordance with the STROBE (Strengthening the Reporting of Observational Studies in Epidemiology) guidelines (Supplementary File 1). Data were collected from ICU of four tertiary hospitals located in Chengdu, Lanzhou and Sanya, China.

Convenience sampling was used to recruit ICU nurses from the participating hospitals. The inclusion criteria were as follows: (a) holding a valid nurse practice certificate and being officially registered; (b) having independently worked in an ICU for at least 1 year; and (c) providing informed consent and voluntarily participating in the study. Nurses who were on vacation, sick leave, or external training during the survey period were excluded. The sample size was estimated using the formula *N* = 4U^2^αS^2^/*δ*^2^ (40). Based on the pilot study, the standard deviation (S) was 0.51 With the significance level set at 0.05 and the allowable error (δ) defined as 0.10, the required sample size was *N* = 4 × 1.96^2^ × 0.51^2^/0.1^2^ ≈ 517. To account for potential sampling bias and invalid responses, a total of 600 questionnaires were distributed. After excluding 16 questionnaires due to insufficient completion time, missing data, or logical inconsistencies, 584 valid questionnaires were retained for analysis. Previous methodological studies indicate that a minimum sample size of 200 is generally adequate for SEM (41, 42). Therefore, the final sample size in this study was sufficient to support SEM analyses.

### 3.2 Data collection

Data were collected between September and November 2025 under the coordination of trained research assistants. Prior to the formal survey, the nursing management departments of the participating hospitals were contacted to obtain institutional permission and to confirm the survey schedule and recruitment procedures. ICU nurses who met the inclusion criteria were then invited to participate. Participants were recruited through hospital mailing lists and WeChat working groups commonly used by ICU nursing staff. The survey was administered using a secure online questionnaire platform. An invitation message containing a brief introduction to the study and the survey link was distributed to potential participants. Nurses were informed that their participation was entirely voluntary and that they could withdraw from the study at any time without any consequences. Before accessing the questionnaire, all participants were presented with an electronic information page outlining the study purpose, procedures, confidentiality assurances, and their rights as participants. Only those who provided electronic informed consent were allowed to proceed to the questionnaire. The survey included items on sociodemographic characteristics, work time pressure, work–family conflict, sleep quality, and burnout.

During data collection, participants were able to contact the research team via email or WeChat if they had any questions regarding the study. To minimize potential response and social desirability biases, anonymity was emphasized, and no identifiable personal information was collected. In addition, recruitment across multiple hospitals was adopted to enhance sample diversity and reduce selection bias.

### 3.3 Measurements

#### 3.3.1 Work time pressure

Work time pressure was operationalized as subjective workload following the approach proposed by Van Emmerik and Jawahar (43). It was measured using their five-item scale, which assesses nurses’ perceived intensity and time-related demands at work. Representative items include “The responsibilities in my work are becoming heavier and heavier” and “I am constantly rushing between different work tasks.” Responses were rated on a five-point Likert scale ranging from 1 (never) to 5 (nearly always). Higher scores indicate greater perceived work time pressure. The scale has demonstrated good reliability and validity in the Chinese cultural context (44). Items were grouped into three parcels using the item-to-construct balance method (45), which were then used as indicators of the latent construct in the SEM. Confirmatory factor analysis (CFA) was conducted to examine the measurement properties of the construct. The measurement model demonstrated an acceptable fit: the chi-square/degree of freedom ratio (*χ*^2^/df) = 2.34, goodness-of-fit index (GFI) = 0.95, adjusted goodness-of-fit index (AGFI) = 0.93, comparative fit index (CFI) = 0.96, incremental fit index (IFI) = 0.96, root mean square error of approximation (RMSEA) = 0.04, and standardized root mean square residual (SRMR) = 0.03. In addition, the scale demonstrated good internal consistency in this study (Cronbach’s *α* = 0.85).

#### 3.3.2 Work–family conflict

Work–family conflict was assessed using the Work–Family Behavioral Role Conflict Scale (WFBRCS), developed by Clark et al. (46) in 2019. The scale is designed to measure individuals’ levels of work–family behavioral role conflict and consists of two dimensions with a total of 30 items, including 15 items assessing the impact of work on family and 15 items assessing the impact of family on work. The scale is structured as a two-factor model representing work-to-family conflict and family-to-work conflict. Representative items include “When work is heavy, I participate less in family communication than usual” and “When my workload is excessive, I talk less with my family after returning home.” All items are rated on a five-point Likert scale ranging from 1 (never) to 5 (very often), with higher scores indicating higher levels of work–family conflict. In this study, the total score was used to reflect overall work–family conflict. The scale has demonstrated good reliability and validity in the Chinese cultural context (47). CFA results revealed a satisfactory construct validity (*χ*^2^/df = 2.56, GFI = 0.98, AGFI = 0.95, CFI = 0.91, IFI = 0.93, RMSEA = 0.06, SRMR = 0.04). In this study, Cronbach’s *α* was 0.87.

#### 3.3.3 Sleep quality

Sleep quality was assessed using the Pittsburgh Sleep Quality Index (PSQI), which is widely used to measure individuals’ subjective sleep quality over the past month (48). The PSQI consists of 19 items and comprises seven components: subjective sleep quality, sleep latency, sleep duration, habitual sleep efficiency, sleep disturbances, use of sleeping medication, and daytime dysfunction. The sum of the seven component scores yields a global score ranging from 0 to 21, with higher scores indicating poorer sleep quality. A global PSQI score greater than 5 is generally considered indicative of poor sleep quality. The PSQI has demonstrated good reliability and validity in the Chinese cultural context (49). Component scores were used as observed indicators in the SEM. CFA results revealed a satisfactory construct validity (*χ*^2^/df = 2.48, GFI = 0.93, AGFI = 0.91, CFI = 0.95, IFI = 0.95, RMSEA = 0.06, SRMR = 0.04). In this study. In this study, the scale demonstrated good internal consistency (Cronbach’s *α* = 0.93).

#### 3.3.4 Burnout

Burnout was measured using the Copenhagen Burnout Inventory (CBI), developed by Kristensen et al. (50). The scale is designed to assess the severity of burnout, primarily focusing on physical and psychological exhaustion, with personal burnout as the core component. The CBI consists of three dimensions: personal burnout (6 items), work-related burnout (7 items), and patient- or colleague-related burnout (6 items), comprising a total of 19 items. All items are rated on a five-point Likert scale ranging from 1 (never), 2 (seldom), 3 (sometimes), 4 (often), to 5 (always). The total score ranges from 19 to 95, with higher scores indicating more severe burnout. The CBI has demonstrated good reliability, with a reported Cronbach’s *α* coefficient of 0.86 (51). Dimension scores were used as observed indicators in the SEM. CFA results revealed a satisfactory construct validity (*χ*^2^/df = 2.41, GFI = 0.92, AGFI = 0.91, CFI = 0.94, IFI = 0.94, RMSEA = 0.06, SRMR = 0.05). In this study, the scale demonstrated good internal consistency (Cronbach’s *α* = 0.86).

### 3.4 Ethical consideration

This study was approved by the Ethics Committee of Northwest Minzu University (Approval No.: XBMU-YX-20260001) and was conducted in accordance with the Declaration of Helsinki.

### 3.5 Data analysis

All statistical analyses were conducted using IBM SPSS Statistics (version 27) and AMOS (version 25). First, descriptive statistics were used to summarize participants’ sociodemographic characteristics and the main study variables. Continuous variables were examined for normality using the Kolmogorov–Smirnov test. Variables with normal distributions were analyzed using Pearson correlation coefficients, whereas Spearman correlation analysis was applied for variables that did not meet normality assumptions. Structural equation modeling based on the covariance structure was employed to test the hypothesized mediation model and the relationships among work time pressure, work–family conflict, sleep quality, and burnout. This approach was selected as it is suitable for theory-driven hypothesis testing and mediation analysis. No post-hoc model modification was performed because the hypothesized model demonstrated acceptable goodness-of-fit indices. Model fit was evaluated using multiple indices, including the *χ*^2^/df, GFI, AGFI, CFI, IFI, RMSEA and SRMR. The following criteria were used to indicate adequate model fit: *χ*^2^/df < 5, GFI > 0.90, AGFI > 0.90, CFI > 0.90, IFI > 0.90, RMSEA ≤ 0.08, and SRMR < 0.05 (41, 42).

To examine the significance of indirect effects, a bootstrapping procedure with 5,000 resamples was conducted. The mediating effects were considered statistically significant if the 95% bias-corrected confidence intervals did not include zero.

## 4 Results

### 4.1 Demographic characteristics

A total of 545 ICU nurses were included in this study. The mean age of the participants was 32.6 years (SD = 6.1), and most were female. The majority of nurses were married and held a bachelor’s degree, with intermediate professional titles being the most common. Most participants worked shifts, with a high proportion reporting frequent night shifts and long weekly working hours. In addition, a substantial proportion of nurses reported having children and primary caregiving responsibilities. Detailed sociodemographic and work-related characteristics are presented in Table 1.

**Table 1:** Descriptions of intensive care nurse characteristics (*N* = 545).

Variables		Frequency (n)	Percentage (%)
Gender	Male	72	13.2
	Female	473	86.8
Age (years)	Mean ± SD	32.6 ± 6.1	–
Marital status	Single	219	40.2
	Married	326	59.8
Education level*	Junior college or below	54	9.9
	Bachelor’s degree	438	80.4
	Master’s or above	53	9.7
Professional title*	Junior	182	33.4
	Intermediate	287	52.7
	Senior	76	13.9
Years of ICU working experience	1–5	175	32.1
	6–10	280	51.4
	>10	90	16.5
Shift work	Yes	497	91.2
	No	48	8.8
Night shift frequency (per month)	≤4 nights	214	39.3
	>4 nights	331	60.7
Number of children	0	203	37.2
	1	267	49.0
	≥2	75	13.8

*Educational level represent years of formal education: junior college or below (≤15 years), bachelor’s degree (16 years), and master’s degree or above (≥18 years); In China, professional titles for nurses are awarded through a formal evaluation system based on clinical experience, professional qualifications, and institutional assessment rather than years of education.

### 4.2 Descriptive statistics and correlation analysis

The descriptive statistics, including mean, standard deviation and correlation coefficients of the study variables, are summarized in Table 2. The mean score of work time pressure was 3.61 (SD = 0.80), while the average scores of work–family conflict, sleep quality, and burnout were 3.66 (SD = 0.53), 9.78 (SD = 1.18), and 3.88 (SD = 0.51), respectively. Normality testing indicated that all continuous variables followed approximately normal distributions, meeting the assumptions for subsequent parametric analyses.

**Table 2:** Descriptive statistics and correlation analysis (*r*).

Variables	1	2	3	4	5	6	7	8	9	10	11	12	13	14	15	M ± SD
1. WTP	1															3.61 ± 0.8
2. WFC	0.310**	1														3.61 ± 0.66
3. FWC	0.277**	0.458**	1													3.71 ± 0.59
4. Work-family conflict	0.344**	0.873**	0.834**	1												3.66 ± 0.53
5. SSQ	0.247**	0.213**	0.177**	0.230**	1											1.58 ± 0.52
6. SL	0.271**	0.183**	0.218**	0.233**	1											1.12 ± 0.41
7. SD	0.295**	0.258**	0.243**	0.293**	0.622**	1										1.25 ± 0.35
8. HSE	0.292**	0.230**	0.184**	0.243**	0.595**	0.616**	1									1.35 ± 0.47
9. SDB	0.270**	0.269**	0.192**	0.272**	0.582**	0.588**	0.667**	1								1.21 ± 0.22
10. USM	0.249**	0.215**	0.199**	0.243**	0.602**	0.594**	0.666**	0.678**	1							1.72 ± 0.55
11. DD	0.309**	0.217**	0.207**	0.247**	0.612**	0.597**	0.637**	0.573**	0.597**	1						1.55 ± 0.52
12. Sleep quality	0.335**	0.275**	0.246**	0.305**	0.826**	0.804**	0.839**	0.817**	0.827**	0.806**	1					9.78 ± 1.18
13. PB	0.400**	0.310**	0.277**	0.344**	0.288**	0.352**	0.286**	0.253**	0.324**	0.273**	0.357**	1				3.85 ± 0.71
14. WRB	0.380**	0.341**	0.270**	0.360**	0.300**	0.302**	0.292**	0.272**	0.283**	0.321**	0.357**	0.427**	1			3.99 ± 0.66
15. CRB	0.352**	0.305**	0.304**	0.356**	0.343**	0.355**	0.314**	0.317**	0.334**	0.320**	0.398**	0.421**	0.421**	1		3.77 ± 0.76
16. Burnout	0.480**	0.407**	0.362**	0.451**	0.396**	0.428**	0.379**	0.359**	0.399**	0.389**	0.472**	0.772**	0.793**	0.788**	1	3.88 ± 0.51

WTP, work time pressure; WFC, work–family conflict; FWC, family–work conflict; SSQ, subjective sleep quality; SL sleep latency; SD, sleep duration; HSE, habitual sleep efficiency; SDB, sleep disturbances; USM, use of sleeping medication; DD, daytime dysfunction; PB, personal burnout; WRB, work-related burnout; CRB, colleague-related burnout. **p < 0.01.

Pearson correlation analysis revealed significant associations among the key variables. Work time pressure was positively correlated with work–family conflict, poor sleep quality, and burnout (*r* = 0.344, 0.335, 0.480, all *p* < 0.01). Work–family conflict showed a significant positive correlation with sleep quality and burnout, and sleep quality was also positively associated with burnout (*r* = 0.305, 0.451, 0.472, all *p* < 0.01). Detailed correlation coefficients are presented in Table 2.

### 4.3 Mediating effect analysis

Structural equation modeling (SEM) was conducted to examine the mediating effects of self-efficacy and organization-based self-esteem in the association between inclusive leadership and job performance. Model fit indices were applied to evaluate the congruence between the hypothesized model and the observed data. The results demonstrated that the proposed model exhibited an acceptable overall fit. Detailed model fit indices are presented in Table 3.

**Table 3:** Model-fitting standard and fitting index of the final model.

Item	*χ*^2^/df	GFI	AGFI	CFI	IFI	RMSEA	SRMR
Model-fitting standard	<5	>0.90	>0.90	>0.90	>0.90	≤0.08	<0.05
Model-fitting index	2.18	0.95	0.94	0.97	0.97	0.07	0.047

**Figure 1:**
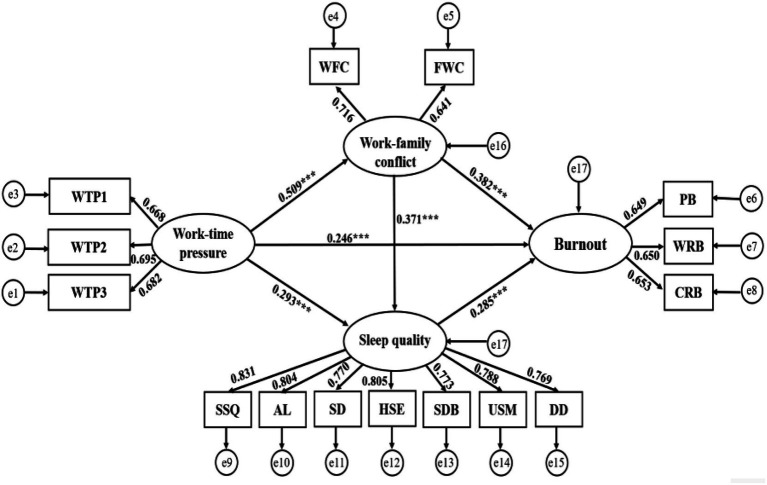
The mediating roles of work–family conflict and sleep quality between work time pressure and burnout. WTP1–WTP3, three parcels of work time pressure; WFC, work–family conflict; FWC, family–work conflict; SSQ, subjective sleep quality; SL sleep latency; SD, sleep duration; HSE, habitual sleep efficiency; SDB, sleep disturbances; USM, use of sleeping medication; DD, daytime dysfunction; PB, personal burnout; WRB, work-related burnout; CRB, colleague-related burnout. ***p < 0.001.

Mediation analysis showed that work time pressure significantly and positively predicted work–family conflict (*β* = 0.509, *t* = 7.307, *p* < 0.001), sleep quality (*β* = 0.293, *t* = 4.436, *p* < 0.001), and burnout (*β* = 0.246, *t* = 3.511, *p* < 0.001). Work–family conflict had a significant positive direct effect on sleep quality (*β* = 0.371, *t* = 5.229, *p* < 0.001) and burnout (*β* = 0.0.382, *t* = 4.898, *p* < 0.001). In addition, sleep quality was found to significantly predict burnout (*β* = 0.285, *t* = 5.264, *p* < 0.001). The standardized path coefficients of the structural model are presented in Figure 1.

To further examine whether work–family conflict and sleep quality mediated the relationship between work time pressure and burnout, indirect effects were tested using a bootstrapping procedure with 5,000 resamples. Both percentile and bias-corrected 95%CI were calculated. Following the recommendations of Preacher and Hayes (52), an indirect effect was considered statistically significant if the corresponding CI did not include 0.

The results indicated that work–family conflict significantly mediated the association between work time pressure and burnout. In addition, sleep quality was found to independently mediate the relationship between work time pressure and burnout. Moreover, a significant serial mediating effect of work–family conflict and sleep quality was identified, suggesting that work time pressure may increase work–family conflict, which in turn impairs sleep quality and ultimately exacerbates burnout among ICU nurses. Detailed results of the mediation analyses are presented in Table 4.

**Table 4:** Bootstrap analysis of the mediating model.

Effect	Path	Standardized *β*	SE	The size of effect	95%CI	
					Lower	Upper
IndA1	WTP → WFC → Burnout	0.194	0.052	33.56%	0.092	0.295
IndA2	WTP → SQ → Burnout	0.084	0.041	14.53%	0.040	0.164
IndA3	WTP → WFC → SQ → Burnout	0.054	0.025	9.34%	0.005	0.103
Direct	WTP → Burnout	0.246				
Total	WTP → Burnout	0.578				

WTP, work time pressure; WFC, work–family conflict; SQ, sleep quality.

## 5 Discussion

The present study examined the relationship between work time pressure and burnout among intensive care unit nurses, as well as the mediating roles of work–family conflict and sleep quality in this association. Specifically, our findings contribute to a deeper understanding of the mechanisms underlying burnout among ICU nurses. First, work time pressure significantly predicted burnout among ICU nurses, indicating that higher perceived time pressure is associated with more severe burnout. Second, mediation analysis revealed that both work–family conflict and sleep quality independently mediated the relationship between work time pressure and burnout. Finally, work–family conflict and sleep quality were found to play a serial mediating role in the association between work time pressure and burnout, suggesting that work time pressure may increase work–family conflict, which subsequently impairs sleep quality and ultimately exacerbates burnout among ICU nurses.

### 5.1 Effect of work time pressure on burnout

In the present study, ICU nurses reported a mean burnout score of 3.88 (SD = 0.51), indicating a moderate-to-high frequency of burnout symptoms, consistent with previous findings in critical care settings (53, 54). Compared with nurses working in general wards, ICU nurses are exposed to sustained high-intensity workloads, frequent emergency situations, and continuous psychological tension, all of which place them at elevated risk for burnout (55). The demanding nature of critical care nursing, characterized by time-sensitive tasks, rapid clinical decision-making, and prolonged vigilance, may partially explain the high burnout levels observed in this study (55).

Consistent with prior research (56), our findings confirmed that work time pressure significantly predicts burnout among ICU nurses, supporting Hypothesis 1, with a moderate direct effect observed in the structural equation model (*β* = 0.246). Work time pressure reflects nurses’ perceptions that available time is insufficient to meet work demands, which represents a core occupational stressor in modern healthcare systems (26). Consistent with the JD–R model described earlier, excessive job demands deplete individuals’ physical and psychological resources, thereby increasing vulnerability to burnout (3). In the ICU environment, nurses must complete complex care tasks within strict time constraints while simultaneously responding to unpredictable emergencies. Persistent exposure to such pressure may lead to chronic fatigue, emotional exhaustion, and reduced psychological resilience (53). Moreover, prolonged work time pressure may limit opportunities for recovery and reflection, accelerating the accumulation of stress and ultimately contributing to burnout (53). These findings underscore the critical role of work time pressure as a structural occupational risk factor for burnout among ICU nurses.

### 5.2 Mediation through work–family conflict

The present study found that ICU nurses reported a mean work–family conflict score of 3.66 (SD = 0.53) on a 5-point Likert scale, indicating a moderate-to-high level of conflict, consistent with previous research showing that nurses in high-intensity clinical environments are particularly vulnerable to work-family role conflicts (20). In the structural model, work–family conflict exhibited a significant association with burnout (*β* = 0.382), suggesting that role-related strain is a substantial contributor to burnout. Moreover, the current results showed that work time pressure not only directly predicted burnout among ICU nurses but also indirectly influenced burnout through the mediating role of work–family conflict, supporting Hypothesis 2.

In previous studies, work–family conflict has often been regarded as a key risk factor for occupational burnout (14, 31). From a theoretical perspective, work–family conflict reflects a form of role stress that arises when the time and energy required by work interfere with family responsibilities (15). Under persistent time pressure, ICU nurses are often forced to extend working hours, sacrifice rest periods, and remain psychologically preoccupied with work, which limits their ability to participate in family life and to recover from occupational stress (15). According to conservation of resources theory, such continuous role interference accelerates the depletion of personal resources, thereby heightening emotional strain and vulnerability to burnout (38). As an affective and interpersonal stressor, work–family conflict may further intensify emotional exhaustion and undermine nurses’ capacity to regulate stress and maintain engagement at work (39). Nurses who experience ongoing conflict between professional and family roles are more likely to develop feelings of guilt, frustration, and helplessness, which can erode professional efficacy and promote depersonalization, ultimately contributing to burnout.

### 5.3 Mediation through sleep quality

In the present study, ICU nurses’ sleep quality was generally poor, which is consistent with previous findings showing that critical care nurses commonly experience sleep disturbances due to rotating shifts, night work, and high psychological demands (13, 57). This indicates that sleep problems are prevalent in ICU settings and remain an important occupational health concern for nurses. In the structural equation model, sleep quality showed a significant association with burnout (*β* = 0.285), indicating that impaired sleep represents a substantial proximal contributor to burnout among ICU nurses. Furthermore, the results demonstrated that work time pressure not only directly predicted burnout but also indirectly affected burnout through the mediating role of sleep quality, supporting Hypothesis 3. This suggests that excessive time pressure contributes to burnout partly by disrupting nurses’ sleep, thereby impairing their physical and psychological recovery. From a mechanistic perspective, persistent work time pressure often leads to extended working hours, irregular schedules, and insufficient rest opportunities, which interfere with circadian rhythms and prolong physiological arousal (58). ICU nurses are frequently exposed to emergencies and emotionally intense situations, making it difficult to disengage from work and achieve restorative sleep. Poor sleep quality reduces the body’s capacity to recover from stress, weakens emotional regulation, and increases vulnerability to fatigue and negative affect (55).

As a result, nurses with chronically impaired sleep are more likely to experience sustained exhaustion, decreased tolerance to stress, and diminished psychological resilience, which accelerate the development of burnout. These findings indicate that sleep quality is a crucial pathway through which work time pressure is translated into burnout, underscoring the importance of sleep-focused interventions and recovery-oriented scheduling in protecting ICU nurses’ occupational well-being.

### 5.4 Mediation through work–family conflict and sleep quality

This study further demonstrated that work–family conflict significantly predicted sleep quality among ICU nurses, which is consistent with previous research showing that heightened inter-role conflict is closely associated with sleep disturbance (39). Moreover, the results indicated that work–family conflict and sleep quality play a sequential mediating role in the association between work time pressure and burnout, supporting Hypothesis 4. ICU nurses are continuously exposed to heavy workloads, frequent emergencies, and high emotional demands, making them particularly vulnerable to role overload and prolonged psychological arousal (20). When nurses experience excessive work time pressure, conflicts between work and family roles are more likely to emerge, increasing emotional strain and cognitive preoccupation (20). Previous studies have shown that individuals with high levels of work–family conflict often have difficulty disengaging from work-related stress, which interferes with relaxation processes and disrupts sleep patterns (38). As work–family conflict intensifies, nurses may experience persistent worry, guilt, and frustration related to unmet family responsibilities, which prolong physiological activation and impair sleep quality (38). Poor sleep further weakens physical recovery, emotional regulation, and stress tolerance (16). Over time, this cascading process-whereby work time pressure heightens work–family conflict, which subsequently impairs sleep-creates a cumulative burden that accelerates emotional exhaustion and contributes to the development of burnout.

## 6 Limitations and future research

This study provides meaningful evidence for understanding the mechanisms linking work time pressure to burnout among ICU nurses and offers important implications for nursing management and occupational health interventions. Nevertheless, several limitations should be acknowledged.

First, this study was conducted in a limited number of hospitals and employed a convenience sampling strategy, which may restrict the generalizability of the findings. Future studies should recruit ICU nurses from multiple regions and healthcare systems and adopt probability-based sampling methods to enhance the representativeness and external validity of the results. Second, all variables were assessed using self-reported measures, which may introduce recall bias and common method variance. In the present study, work time pressure was operationalized using a subjective workload scale intended to capture perceived work intensity and time-related demands. However, some items may also reflect broader workload or job strain rather than time insufficiency alone, which may partially inflate its association with burnout. Future studies should employ more specific measures of time pressure or multi-method approaches to improve construct differentiation. Third, demographic and work-related characteristics (e.g., age, shift work status, and family responsibilities) were not included as covariates in the structural equation model. These factors may influence sleep quality and burnout and could confound the observed relationships. Future research should incorporate relevant control variables to further test the robustness of the model.

Finally, the cross-sectional design limits the ability to infer causal relationships among work time pressure, work–family conflict, sleep quality, and burnout. Although the proposed serial mediation model was theoretically grounded, the directionality of these relationships cannot be definitively established, and alternative models (e.g., sleep disturbances influencing work–family conflict or burnout affecting work time pressure) may also plausibly fit the data. Longitudinal or prospective studies are therefore needed to examine the temporal and dynamic interplay among these variables and to verify the proposed mediating mechanisms over time.

## 7 Conclusion

In the context of increasing clinical demands and persistent staffing shortages in critical care, burnout among ICU nurses has become a pressing concern. This study found that work time pressure was associated with burnout and was indirectly related to burnout through patterns consistent with the sequential mediating roles of work–family conflict and sleep quality. These findings provide insight into a potential psychosocial–physiological pathway linking time demands to burnout and may help inform time-management, family-supportive, and recovery-oriented strategies to protect ICU nurses’ well-being and promote a sustainable critical care workforce.

## 8 Implications for clinical practice

The findings of this study offer valuable insights for nursing management and clinical practice, particularly in intensive care settings characterized by high workload and sustained time pressure. First, the significant association between work time pressure and burnout suggests the potential importance of examining staffing models, shift arrangements, and workload distribution to reduce excessive time demands on ICU nurses. Strategies such as flexible scheduling, adequate nurse–patient ratios, and protected rest periods may be considered as possible approaches to mitigating chronic time pressure. Second, the mediating role of work–family conflict highlights the potential relevance of organizational policies that support work–life balance. Nurse managers may consider family-supportive practices, including predictable scheduling, opportunities for shift exchanges, and access to family-friendly resources, to help nurses better manage competing work and family roles. Third, the association between sleep quality and burnout underscores sleep health as a potentially important area for occupational well-being. Healthcare institutions could incorporate sleep promotion strategies into occupational health programs, such as optimizing shift rotations, providing fatigue management education, and creating rest-friendly environments.

Finally, the serial mediation pathway suggests that burnout may be influenced by multiple interconnected factors. Therefore, comprehensive and multifaceted organizational approaches that address time demands, work–family balance, and sleep health may contribute to improving nurses’ well-being. However, longitudinal and interventional studies are needed to determine whether modifying these factors would effectively reduce burnout.

## Data Availability

The raw data supporting the conclusions of this article will be made available by the authors, without undue reservation.
